# Reverse Engineering of Gene Regulatory Networks: A Comparative Study

**DOI:** 10.1155/2009/617281

**Published:** 2009-04-22

**Authors:** Hendrik Hache, Hans Lehrach, Ralf Herwig

**Affiliations:** 1 Vertebrate Genomics-Bioinformatics Group, Max Planck Institute for Molecular Genetics, IhnestraÃŸe 63-73, 14195 Berlin, Germany

## Abstract

Reverse engineering of gene regulatory networks has been an intensively studied topic in bioinformatics since it constitutes an intermediate step from explorative to causative gene expression analysis. Many methods have been proposed through recent years leading to a wide range of mathematical approaches. In practice, different mathematical approaches will generate different resulting network structures, thus, it is very important for users to assess the performance of these algorithms. We have conducted a comparative study with six different reverse engineering methods, including relevance networks, neural networks, and Bayesian networks. Our approach consists of the generation of defined benchmark data, the analysis of these data with the different methods, and the assessment of algorithmic performances by statistical analyses. Performance was judged by network size and noise levels. The results of the comparative study highlight the neural network approach as best performing method among those under study.

## 1. Introduction

Deciphering the complex structure of transcriptional regulation of gene expression by means of computational methods is a challenging task emerged in the last decades. Large-scale experiments, not only gene expression measurements from microarrays but also promoter sequence searches for transcription factor binding sites and investigations of protein-DNA interactions, have spawned various computational approaches to infer the underlying gene regulatory networks (GRNs). Identifying interactions yields to an understanding of the topology of GRNs and, ultimately, of the molecular role, of each gene. On the basis of such networks computer models of cellular systems are set up and in silico experiments can be performed to test hypotheses and generate predictions on different states of these networks. Furthermore, an investigation of the system behavior under different conditions is possible [1]. Therefore reverse engineering can be considered as an intermediate step from bioinformatics to systems biology.

The basic assumption of most reverse engineering algorithms is that causality of transcriptional regulation can be inferred from changes in mRNA expression profiles. One is interested in identifying the regulatory components of the expression of each gene. Transcription factors bind to specific parts of DNA in the promoter region of a gene and, thus, effect the transcription of the gene. They can activate, enhance, or inhibit the transcription. Changes of abundances of transcription factors cause changes in the amount of transcripts of their target genes. This process is highly complex and interactions between transcription factors result in a more interwoven regulatory network. Besides the transcription factor level, transcriptional regulation can be affected as well on DNA and mRNA levels, for example, by chemical and structural modifications of DNA or by blocking the translation of mRNAs by microRNAs [2]. Usually these additional regulation levels are neglected or included as hidden factors in diverse gene regulatory models. Unfortunately, data on protein concentration measurements are currently not available in a sufficient quantity for incorporation in reverse engineering analysis. Therefore, gene expression profiles are most widely used as input for these algorithms. Probably this will change in future reverse engineering research.

Several reverse engineering methods were proposed in recent years which are based on different mathematical models, such as Boolean networks [3], linear models [4], differential equations [5], association networks [6, 7], static Bayesian networks [8], neural networks [9], state space models [10, 11], and dynamic Bayesian networks [12–14]. There are static or dynamic, continuous or discrete, linear or nonlinear, deterministic or stochastic models. They can differ in the information they provide and, thus, have to be interpreted differently. Some methods result in correlation measures of genes, some calculate conditional independencies, and others infer regulation strengths. These results can be visualized as directed or undirected graphs representing the inferred GRNs. For that, a discretization of the results is necessary for some methods. Each concept has certain advantages and disadvantages. A historical perspective of different methods applied until 2002 is given by van Someren et al. [15]. de Jong [16] and more recently Gardner and Faith [17] discuss further details and mathematical aspects.

In order to perform a comparative study we have chosen six reverse engineering methods proposed in literature based on different mathematical models. We were interested in applications for the analysis of time series. The methods should be freely downloadable, easy in use, and having only a few parameters to adjust. We included two relevance network methods; the application ARACNe by Basso et al. [6], which is based on mutual information and the package ParCorA by de la Fuente et al. [18], which calculates partial Pearson and Spearman correlation of different orders. Further, the neural network approach GNRevealer by Hache et al. [9] is compared. As an example for a Bayesian approach, the Java package Banjo [13] for dynamic models is employed. The state space model LDST proposed by Rangel et al. [10] and a graphical Gaussian model by Schäfer and Strimmer [7] in the GeneNet package are as well included in our study. We implemented the applications in a reverse engineering framework starting with artificially generated data to compare the different applications under the same conditions.

Artificial data has been used because validation and comparison of performances of algorithms have to be accomplished under controlled conditions. It would have been desirable to include experimentally determined gold standard networks that represent the knowledge of all interactions validated by single or multiple experiments. Unfortunately, there are not enough gold standard networks and appropriate experimental data available for a large comparative study. For such a study one needs a sufficiently large amount of data of different sizes, different types, that is, steady state or time series, from different experiments, for example, overexpression, perturbation, or knockdown experiments. Therefore we performed in silico experiments to obtain the required data for our performance tests.

Quackenbush [19] pointed out, that the use of artificially generated data can help to provide an understanding of how data are handled and interpreted by various methods, albeit the datasets usually do not reflect the complexity of real biological data. Their analysis involved various clustering methods. The application to synthetic datasets by computational methods is as well proposed by Mendes et al. [20] for objective comparisons. Repsilber and Kim [21] followed also the approach of using simulated data and presented a framework for testing microarray data analysis tools.

An artificial data generator has to be independent of the reverse engineering algorithms to avoid a bias in the test results. In addition, the underlying artificial GRN of a data generator has to capture certain features of real biological networks, such as the scale-free property. For this study we used the web application GeNGe [22] for the generation of scale-free networks with an mRNA and protein layer with nonlinear dynamics and performed in silico perturbation experiments.

Having specified artificial networks the computed and the true networks can be compared and algorithmic performance can be assessed with statistical measures. We used various measures in this study, such as a sensitivity, specificity, precision, distance measure, receiver operator characteristic (ROC) curves, and the area under ROC curves (AUCs).

By means of these measures we characterized the reverse engineering method performances. It is shown that the sensitivity, specificity, and precision of all analyzed methods are low under the condition of this study. Averaged over all results, the neural network approach shows the best performances. In contrast, the Bayesian network approaches identified only a few interactions correctly. We tested different sets of data, including different sizes and noises to highlight the conditions for better performances of each method.

## 2. Methods and Applications

A variety of reverse engineering methods has been proposed in recent years. Usually a computational method is based on a mathematical model with a set of parameters. These model specific parameters have to be fitted to experimental data. The models vary from a more abstract to a very detailed description of gene regulation. They can be static or dynamic, continuous or discrete, linear or nonlinear, deterministic or stochastic. An appropriate learning technique has to be chosen for each model to find the best fitting network and parameters by analyzing the data. Besides these model driven approaches, for example, followed by Bayesian networks and neural networks, there are statistical approaches to identify gene regulations, for example, relevance networks.

For this study we have chosen reverse engineering applications which belong to one of the following classes: relevance networks, graphical Gaussian models, Bayesian networks, or neural networks. In this section we will give an overview of the basic models and discuss the applications we used. All software can be downloaded or obtained from the algorithm developers. An overview is given in Table 1. 

**Table 1:** Reverse engineering applications used in this study.

Name	Type	Info	Reference
ARACNe	relevance network with mutual information	C command line	Basso et al. [6]
ParCorA	relevance network with partial Pearson or Spearman correlation	C command line	de la Fuente et al. [18]
GNRevealer	neural network	C++ command line	Hache et al. [9]
Banjo	Bayesian network	Java command line	Yu et al. [13]
LDST	state space model	Matlab script	Rangel et al. [10]
GeneNet	graphical Gaussian model	R script	Schäfer and Strimmer [7]

The applications can be downloaded or obtained from the algorithm developers. See references for more details.

### 2.1. Relvance Networks

Methods based on relevance networks are statistical approaches that identify dependencies or similarities between genes across their expression profiles. They do not incorporate a specific model of gene regulation. In a first step correlation is calculated for each pair of genes based on different measures, such as Pearson correlation, Spearman correlation, and mutual information. The widely used Pearson correlation indicates the strength of a linear relationship between the genes. In contrast to that Spearman's rank correlation can detect nonlinear correlations as well as mutual information. It is assumed that a nonzero correlation value implies a biological relationship between the corresponding genes. The algorithm ARACNe developed by Basso et al. [6] uses the Data Processing Inequality (DPI) for that purpose. In each triplet of fully connected nodes in the network obtained after the first step, the edges with the lowest mutual information will be removed. In contrast, de la Fuente et al. [18] use partial correlations in their proposed method to eliminate indirect interactions. A partial correlation coefficient measures the correlation between two genes conditioning on one or several other genes. The number of genes conditioning the correlation determines the order of the partial correlation. In the program package ParCorA by de la Fuente et al. [18] the partial correlations up to 3rd order for Pearson and 2nd order for Spearman correlation are implemented. We compared all provided correlation measures.

An inferred network from a relevance network method is undirected by nature. Furthermore, statistical independence of each data sample is assumed, that is, that measurements of gene expression at different time points are assumed to be independent. This assumption ignores the dependencies between time points. Nevertheless, we applied these methods on simulated time series data to study the predictive power of these approaches.

### 2.2. Graphical Gaussian Models

Graphical Gaussian models are frequently used to describe gene association networks. They are undirected probabilistic graphical models that allow to distinguish direct from indirect interactions. Graphical Gaussian models behave similar as the widely used Bayesian networks. They provide conditional independence relations between each gene pair. But in contrast to Bayesian networks graphical Gaussian models do not infer causality of a regulation.Graphical Gaussian models use partial correlation conditioned on all remaining genes in the network as a measure of conditional independence. Under the assumption of a multivariate normal distribution of the data the partial correlation matrix is related to the inverse of the covariance matrix of the data. Therefore the covariance matrix has to be estimated from the given data and to be inverted. From that the partial correlations can be determined. Afterwards a statistical significance test of each nonzero partial correlation is employed.

We used the graphical Gaussian implementation GeneNet by Schäfer and Strimmer [7]. It is a framework for small-sample inference with a novel point estimator of the covariance matrix. An empirical Bayes approach to detect statistically significant edges is applied to the calculated partial correlations.

### 2.3. Neural Networks

A neural network can be considered as a model for gene regulation where each node in the network is associated with a particular gene. The value of the node is the corresponding gene expression value. A directed edge between nodes represents a regulatory interaction with a certain strength indicated by the edge weight. The dynamic of a time-discrete neural network of  nodes is described by a system of nonlinear update rules for each node value : (1)

The parameters of the model are the weights , where  represents the influence of node  on node , activation strengths , bias parameters , and degradation rates . The effects of all regulating nodes are added up and have a combined effect on the connected node. The sigmoidal activation function  realizes a saturation of the regulation strength. Self-regulation and degradation are implemented in the mathematical model as well.

A learning strategy for the parameters is the Backpropagation through time (BPTT) algorithm described by Werbos [23] and applied to genetic data by Hache et al. [9]. The BPTT algorithm is an iterative, gradient-based parameter learning method which minimizes the error function: (2)

by varying the parameters of the model () during every iteration step.  is the computed values vector and the values  are the given expression data of the mRNAs at discrete time points. The computed matrix  of regulation strength is a matrix of real values, which has to be discretized to obtain a binary or ternary matrix, representing, ultimately, the topology.

### 2.4. Dynamic Bayesian Networks

A Bayesian network is a stochastic probabilistic graphical network model defined by a directed acyclic graph (DAG) which represents the topology and a family of conditional probability distributions. In contrast to other models nodes represent random variables and edges conditional dependence relations between these random variables. A dynamic Bayesian network is an unfolded static Bayesian network over discrete time steps. Assuming that nodes are only dependent of direct parents in the previous time layer, the joint probability distribution of a dynamic Bayesian network can be factorized: (3)

where  is the set of random variables  with value  for each node  at time .  represents the set of parents of node  in the previous time slice . The temporal process is Markovian and homogeneous in time, that means a variable  is only dependent of parents at the time point  and the conditional distribution does not change over time, respectively.

For discrete random variables the conditional probability distributions can be multinomial. With such a distribution nonlinear regulations can be modeled, but a discretization of continuous data is needed. The number of parameters in such a model increases exponentially with the number of parents per node. Therefore, this number is often restricted by a maximum. The program package Banjo by Yu et al. [13], which we used in this study as an representative for a Bayesian method, follows a heuristic search approach. It seeks in the network space for the network graph with the best score, based on the Bayesian Dirichlet equivalent (BDe) score. A score here is a statistical criterion for model selection. It can be based on the marginal likelihood  for a dataset  given a graph structure . The BDe score is a closed form solution for the integration of marginal likelihood, derived under the assumption of a multinomial distribution with a Dirichlet prior. See, for example, Heckerman et al. [24] for more details. It requires discrete values as input. A discretization is performed by the program. For that, two methods are provided; interval and quantile discretization. The number of discretization levels can be specified as well. We used the quantile discretization with five levels. The output network of Banjo is a signed directed graph.

### 2.5. State Space Models

A further reverse engineering approach is a state space model. They constitute a class of dynamic Bayesian networks where it is assumed that the observed measurements depend on some hidden state variables. These hidden variables capture the information of unmeasured variables or effects, such as regulating proteins, excluded genes in the experiments, degradations, external signals, or biological noise.

A state space model is proposed by Schäfer and Strimmer [7]. The model for gene expression includes crosslinks from an observational layer to a hidden layer: (4)

Here,  denotes the gene expression levels at time  and  the unobserved hidden factors. The matrix  captures gene-gene expression level influences at consecutive time points and the matrix  denotes the influence of the hidden variables on gene expression level at each time point. Matrix  models the influence of gene expression values from previous time points on the hidden states and  is the state dynamics matrix. The matrix  has to be determined, which captures not only the direct gene-gene interactions but also the regulation through hidden states over time. A nonzero matrix element  denotes activation or inhibition of gene  on gene  depending on its sign.

## 3. Data

For the comparative study of reverse engineering methods we generated a large amount of expression profiles from various GRNs and different datasets. We performed in silico perturbation experiments by varying the initial conditions randomly within the network and data generator GeNGe [22]. A discretization step is followed if required by the reverse engineering application internally, for example, by DBN with a quantile discretization.

In a first step we generated random scale-free networks in GeNGe to obtain GRNs of different sizes. Directed scale-free networks are generated in GeNGe with an algorithm proposed by Bollobás et al. [25], for each generated network a mathematical model of gene regulation is constructed. We assembled a two-layer system, with an mRNA and a protein layer. The kinetics of the concentration of an mRNA and protein pair, associated to an arbitrary gene, are described by (5)

where  and  are the maximal transcription rate of the mRNA and translation rate of the corresponding protein, respectively.  and  are the degradation rates.  is dependent of  concentrations  of the proteins acting as transcription factors of the gene. A transcription factor is indexed by . Note that all the parameters, , the transcription function , and the set  of transcription factor indices are gene specific and can vary between genes.

In the GRN models we used the logic described by Schilstra and Nehaniv [26] for the transcription kinetics . We distinguish between input genes, which have no regulatory elements in the model and regulated genes, which have at least one regulator. Input genes have a constant linear production. In contrast, regulated genes have no such production. They can only be expressed, if a regulator bounds to the corresponding promoter region of the DNA. Therefore, the transcription factors are essential for the expression of such genes. Other kinetic schemata are also conceivable but not considered here. With the assumption of noncompetitive binding and an individual, gene-dependent regulation strengths  of each transcription factor  of the gene, we derived the kinetic law: (6)

A regulation strength  of transcription factor  stands for activation and  for inhibition of the gene's transcription. The second term in the first case of (6) implements the assumption that regulated genes do not have a constant production rate. In each generated network we set 70% of all regulators as activators and the others as inhibitors. This ratio is arbitrarily chosen, but is motivated by the network proposed by Davidson et al. [27], where more activators than inhibitors can be found. The regulation strengths  are randomly chosen from a uniform distribution over the interval  and  for activators and inhibitors, respectively.

Time series of mRNAs are obtained by first drawing randomly the initial concentrations of each component of the model from a normal distribution with the steady state value of this component as mean and  as coefficient of variation. Steady states are determined numerically in sufficiently long presimulations where changes of concentrations did not anymore occur. The simulations are then performed using the initial conditions. With this approach we simulated global perturbations of the system. We inspected the time series and selected all time series which show similar behavior, that is, relaxation in the same steady state over time. From the simulated mRNA values we picked  values at different time steps during the relaxation of the system as the input data of all reverse engineering algorithms. Note that all values are in an arbitrary unit system.

To simulate experimental errors we added Gaussian noise with different coefficient of variations (cvs) to each expression value in a final step of data generation. The mean of the Gaussian distribution is the unperturbed value. The cv represents the level of noise.

We investigated the impact of different numbers of time series of mRNAs and noise levels on the reconstruction results. For this study we generated randomly five networks of sizes 5, 10, 20, and 30 nodes each. For each network we simulated 5, 10, 20, 30, and 50 time series by repeating the simulation accordingly with different initial values, as described above. For a network of size ten and ten time series, the data matrix contains 500 values (10 nodes   10 time series   5 time points). We added to the profiles noise with cvs equal to , , , , , and . After that we took from each time series five equidistant time points in the region, where changes in the expression profiles occur. Hence, each reverse engineering application had to analyze 600 datasets ( network sizes   5 time series sets   6 noise levels). All datasets and models are provided as supplementary material.

## 4. Postprocessing

**Figure 1:**
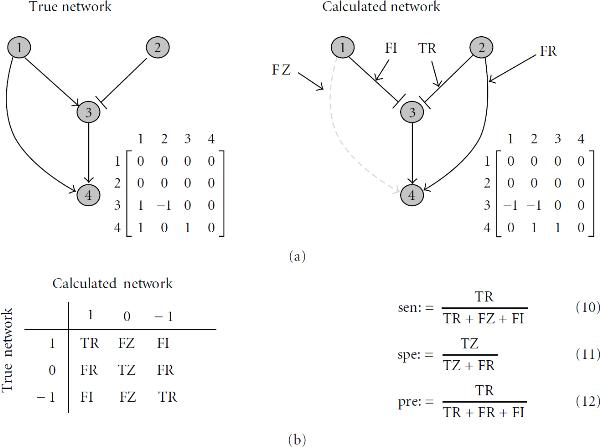
**Definitions**. (a) Example of a true (model) network and a calculated network. The adjacency matrix represents the network structure. (b) *Left:* gene regulatory models have three discrete states (: activation, : inhibition, : nonregulation). We consider the kind of regulation (activation or inhibition) in the classification of the results according to the models: TR: True regulation; TZ: True zero; FR: False regulation; FZ: False zero; FI: False interaction. *Right:* definitions for sensitivity, (10), specificity, (11), and precision, (12).

For all results of the relevance network, graphical Gaussian model, and neural network approaches we performed a postprocess to obtain a resulting network. Most of the entries in the resulting matrices are unequal to zero. This represents a nearly fully connected graph. In contrast the true input networks are sparse. Hence, we discretized each output matrix, representing the correlations or regulation weights between the genes, using an optimized threshold for each method. Such threshold minimizes the distance measure: (7)

where  is the sensitivity and  the specificity. See Figure 1 for definitions. A distance of zero is optimal. We considered all 600 results for this optimization strategy. The sensitivity and specificity are the averaged values over all reconstruction results and are equally weighted, that is, the distance is a balance between calculated true regulations and true zeros (nonregulations) among all regulations and non-regulations, respectively, in the model. A lower threshold would result in more true regulations but with more false regulations and less true zeros, that is, the sensitivity is increased while the specificity is decreased. A higher value has the opposite effect.

## 5. Validation

For the validation, we calculated the sensitivity, specificity, and precision as defined in Figure 1. Sensitivity is the fraction of the number of found true regulations to all regulations in the model. Specificity defines the fraction of correctly found noninteractions to all noninteractions in the model. Since the number of noninteractions in the model is usually large compared to false regulations, the specificity is then around one and does not give much information about the quality of the method. Therefore, we calculated as well precision, which is the fraction of the number of correctly found regulations to all found regulations in the result.

The relevance network and graphical Gaussian approaches give no information about the direction of a link. Only undirected graphs can be revealed. Therefore, we used modified definitions for sensitivity , specificity , and precision  that consider a regulation from node  to  in the resulted network as true, if there is a link from node  to  or  to  in the model network, that is, the network is assumed as undirected.

Further we calculated a measure which considers an undirected graph and additionally does not count false interactions, that is, false identified activations or inhibitions. The corresponding networks are assumed as undirected with no interactions type, that is, these are undirected, binary graphs. Equations (10), (11), and (12) are reduced then to the usual definition of sensitivity and specificity, respectively. The modified measures are denoted with , , .

To obtain a single value measure for one result we calculated the combined measure defined in (7). This distance measure  combines the sensitivity and specificity equally weighted to a single value measure. Low values indicate good reconstruction performances. Correspondingly to sensitivity and specificity, the undirected distance measures are indicated by  and the binary, undirected measure by .

Rather than selecting an arbitrary threshold for discretizing the resulting matrices it is convenient to use the curves of sensitivity versus specificity or precision versus recall for thresholds in interval  to assess the method performances. The measure recall is equal to the sensitivity. These curves are called receiver operator characteristics (ROCs). To obtain a single value measure one can use the area under the curve (AUC). We calculated AUC of the sensitivity versus specificity curves as an additional performance measure. Larger values indicate better performances. Note that a value less than 0.5 does not mean anticorrelation, since a random classifier is not represented by the diagonal.

## 6. Performance Results

We accomplished a systematic evaluation of the performances of six different reverse engineering applications using artificial gene expression data. In the program package ParCorA there are seven correlation measures implemented, including Pearson and Spearman correlation of different orders, which we all used. 600 datasets, with different numbers of genes, dataset sizes, and noise levels, were analyzed by each of the total twelve applications.

For all relevance network methods, graphical Gaussian model, and neural network we determined an optimized threshold for discretization of the results considering all datasets. The thresholds are listed in Table 2. 

**Table 2:** Discretization thresholds for different types of measures

Application	Threshold		
GNRevealer (neural network) [NN]			
GeneNet (graphical Gaussian model) [GGM]	—	0.02	
Partial Pearson correlation, 0th order [PC0]	—		
Partial Pearson correlation, 1st order [PC1]	—		
Partial Pearson correlation, 2nd order [PC2]	—		
Partial Pearson correlation, 3rd order [PC3]	—		
Partial Spearman correlation, 0th order [SC0]	—		
Partial Spearman correlation, 1st order [SC1]	—		
Partial Spearman correlation, 2nd order [SC2]	—		

The averaged reconstruction performances over all datasets with regard to different validation measures are given in Table 3. Since some applications, such as relevance networks give no information about the direction of regulation, we calculated as well undirected measures, denoted with . Additionally, we computed measures, which considers undirected results and neglects the kind of interaction information (activation or inhibition). These measures are indicated by .

**Table 3:** Performance results

Name	Type	sen	spe	pre		AUC
DBN	D	0.030(0.084)	0.953(0.117)	0.041(0.119)	0.971(0.067)	—
	U	0.050(0.119)	0.924(0.173)	0.064(0.138)	0.953(0.083)	—
	B	0.084(0.196)	0.924(0.173)	0.099(0.193)	0.919(0.116)	—
NN	D	**0.276(0.197)**	0.660(0.216)	**0.091(0.073)**	**0.800(0.131)**	**0.324**
	U	**0.334(0.204)**	0.617(0.278)	**0.208(0.162)**	**0.768(0.157)**	**0.350**
	B	0.539(0.255)	0.574(0.278)	**0.281(0.164)**	**0.628(0.147)**	**0.557**
SSM	D	0.027(0.073)	**0.973(0.053)**	0.052(0.139)	0.973(0.068)	—
	U	0.030(0.075)	**0.975(0.048)**	0.094(0.224)	0.970(0.071)	—
	B	0.049(0.114)	**0.975(0.048)**	0.153(0.297)	0.951(0.110)	—
GGM	D	—	—	—	—	—
	U	0.238(0.198)	0.585(0.290)	0.116(0.157)	0.868(0.145)	0.266
	B	0.442(0.326)	0.585(0.290)	0.225(0.212)	0.695(0.185)	0.526
MI	D	—	—	—	—	—
	U	0.196(0.129)	0.745(0.146)	0.163(0.124)	0.843(0.130)	—
	B	0.287(0.177)	0.745(0.146)	0.239(0.170)	0.757(0.162)	—
PC0	D	—	—	—	—	—
	U	0.177(0.154)	0.659(0.234)	0.106(0.153)	0.891(0.116)	0.253
	B	0.513(0.228)	0.492(0.223)	0.220(0.174)	0.703(0.132)	0.506
PC1	D	—	—	—	—	—
	U	0.228(0.161)	0.541(0.226)	0.105(0.131)	0.898(0.126)	0.249
	B	**0.545(0.225)**	0.461(0.214)	0.219(0.168)	0.705(0.135)	0.502
PC2	D	—	—	—	—	—
	U	0.186(0.136)	0.635(0.154)	0.108(0.125)	0.892(0.125)	0.249
	B	0.493(0.186)	0.515(0.151)	0.217(0.157)	0.702(0.140)	0.504
PC3	D	—	—	—	—	—
	U	0.221(0.154)	0.573(0.179)	0.108(0.102)	0.888(0.135)	0.250
	B	0.526(0.223)	0.484(0.190)	0.217(0.142)	0.701(0.130)	0.506
SC0	D	—	—	—	—	—
	U	0.285(0.195)	0.555(0.217)	0.129(0.135)	0.842(0.141)	0.298
	B	0.491(0.247)	0.555(0.217)	0.230(0.175)	0.676(0.139)	0.528
SC1	D	—	—	—	—	—
	U	0.272(0.186)	0.563(0.215)	0.127(0.135)	0.849(0.137)	0.291
	B	0.518(0.233)	0.518(0.209)	0.231(0.173)	0.682(0.135)	0.521
SC2	D	—	—	—	—	—
	U	0.256(0.165)	0.588(0.143)	0.125(0.117)	0.851(0.137)	0.290
	B	0.500(0.188)	0.542(0.145)	0.229(0.155)	0.678(0.129)	0.523

Results of each application applied on all 600 datasets. DBN: Dynamic Bayesian network; NN: Neural network; MI: ARACNE; PC: Partial correlation with Pearson correlation of given order; SC: Partial correlation with Spearman correlation of given order; SSM: State space model; GGM: Graphical Gaussian model. Type is the performance measure type, that can be D for directed graph, U for undirected graph, B for binary and undirected graph. sen, spe, pre, and d are defined in (10), (11), (12), and (7), respectively. AUC is the area under the ROC curve. The averaged values are given with standard deviation in parenthesis. The top value of each type and column is highlighted in boldface.

None of the reconstruction methods outperforms all other methods. Further, no method is capable of reconstructing the entire true network structure for all datasets. In particular sensitivity and precision are low for all methods. A low precision means that among the predicted regulations, there are only a few true regulations. In the study the precision is always lower than 0.3. This is due to the fact that several input datasets carry a high error level. For example, the input data includes time series with noise up to 50% (). This can bias the performance results. On the other side, the dataset contains small scale time series (5 genes) with up to 50 repetitions and performances are much better with respect to these data (data not shown).

The neural network approach shows the best results among the algorithms tested with regard to the distance measures  and AUC. On average it identifies over 27% of the directed regulations correctly, the highest value among all methods. This is remarkable considering the high error level inherent in several datasets. However, simultaneously the specificity is quite low. That indicates that many false regulations were identified. Less than 10% of the found regulations are true (precision). In contrast, the Bayesian network approaches, DBN and SSM, have a large specificity but with a very low sensitivity. Hence the performances are poor. Only a few regulations were identified and only some of them are true (low precision).

The relevance network approaches using partial Spearman rank correlation show better performances compared to partial Pearson correlation with regard to the distance measure and AUC. This might be explainable by the robustness of the Spearman correlation taking ranks into account rather than actual expression data which is advantageous inspite of noisy data. Surprisingly, with higher orders of partial Pearson and Spearman correlation the distance measures  are not increasing. It is around  for Pearson and  for Spearman correlation. However, with in average up to 55% () of true undirected links could be identified by 1st-order Pearson correlation, neglecting the type of interactions. But 0th-order Spearman correlation identified over 55% (in average) of all nonregulations.

The MI method (ARACNE) found the fewest true undirected links (low sensitivity ), except the DBN and SSM methods. In comparison to the relevance network approaches, MI has a considerably larger specificity , that is, MI identifies more nonregulations in the network correctly (true zeros). GGM shows the opposite behavior. It has a larger sensitivity but a lower specificity compared to MI.

**Figure 2:**
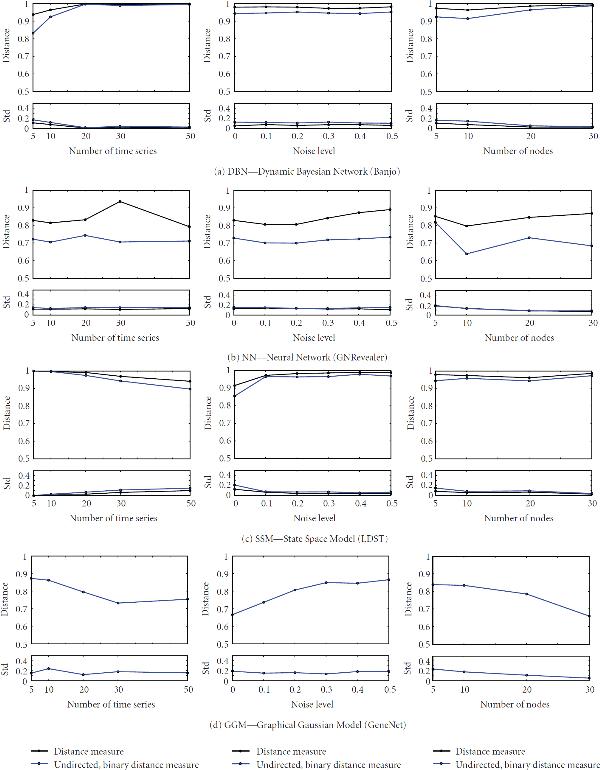
**Performances of applications**. The directed distance measure  (black line) and undirected, binary distance measures  (blue line) is plotted with standard deviations below. The measure  is not available for all methods. From left to right in each row: distance measures over number of time series, cv, and network sizes. Each value is an average over all results with the given feature of the abscissa (see text for more details). A smaller distance indicates a better performance.

**Figure 3:**
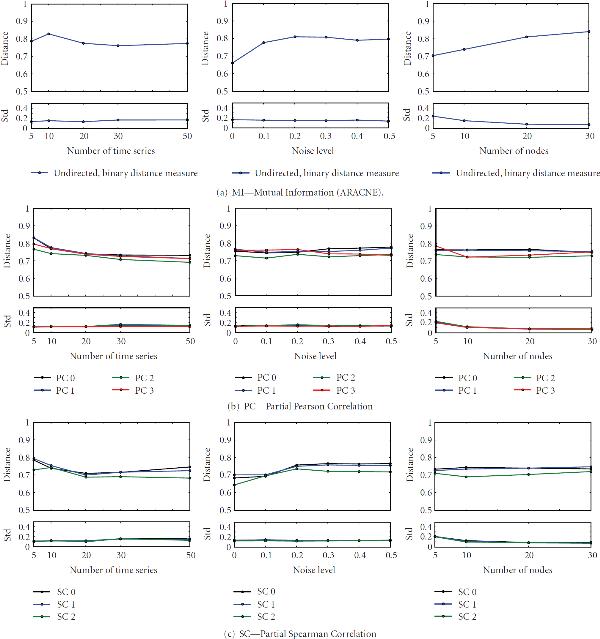
**Performance of applications**. See also Figure 2. Only the undirected, binary distance measure  is plotted for partial Pearson and partial Spearman correlations. Colors indicate the different correlation measures.

In Figures 2 and 3 more details about the performance of each method are plotted with the error resulting from the averaging. The performance behavior with regard to different number of time series, that is, size of dataset, different noise levels, that is, coefficient of variation, and network size, that is, different number of nodes is shown. The distance measures over the number of time series were averaged over five different networks with four different sizes and six different noise levels, that is, in total of 120 datasets. In case of cv and network size, values were averaged over results from 100 and 150 datasets, respectively.

An overall trend is seen for increasing coefficient of variations. As expected the performances of each method decreases with increasing cv (middle column). Though, the distance measures for Banjo does change only slightly, it remains on a very large value. This indicates a poor reconstruction performance. SSM shows a similar behavior. The distance measure  increases very fast for the graphical Gaussian model (GGM). However, the values of the measure decrease noticeable with the size of network. This is in contrast to all other methods, where for larger networks a decrease of reconstruction performance is observable. Surprisingly, the dataset size, that is, the number of time series does not have a large impact on all methods, except for SSM, where the distance measure decreases from a high value. However, in general, more available data would not always result in a better performance.

Among all partial Pearson correlations methods, the 2nd order outperforms the others. It has a slightly better performance measure under all conditions. This is similar to the 2nd order of partial Spearman correlation. It shows the best performance in all plots. Further, it is always below the best partial Pearson correlation.

## 7. Discussion and Conclusion

The comparative study shows that the performances of the reverse engineering methods tested here are still not good enough for practical applications with large networks. Sensitivity, specificity, and precision are always low. Some methods predict only few gene interactions, such as DBN, indicated by a low sensitivity and, in contrast to that, other methods identify many false regulations, such as the correlation measures. We tested different sets of data, including different sizes and noises to highlight the conditions for better performances of each method.

DBN performs poorly on all datasets. Under no condition of the study it shows an appropriate performance. The specificity is always very large, but with a very low sensitivity. Only very few regulations were identified and the performance does not improve with larger datasets. It is known that Banjo requires large datasets for better performances [13]. This may be a reason for the observations. A similar behavior shows the other Bayesian network approach, the state space model. It is slightly better than DBN, but SSM has as well very low sensitivity. The predictive power of such a stochastic approach could not be shown under the conditions in this study.

The neural network approach shows the best results among all methods tested. It has a balance between true positives and true zeros. This is due to the appropriately chosen threshold for the postprocess discretization. Nevertheless, NN predicts many regulations and many of them are incorrect, that is, it has many false regulations. Even with a large number of datasets, a complete reconstruction is not possible.

Schäfer and Strimmer [7], the authors of the GeneNet package including GGM, pointed out that their method is intended for analysis of small sample sizes. It requires a large number of genes in the dataset to estimate the null distribution from the data, which is used for detecting statistical significant interactions. This behavior is shown in Figure 2. Increasing number of genes in the dataset decrease the distance measure  resulting in a better performance.

The assumption of statistical independence of each time point measurement is not satisfied, although ARACNE performs not all that bad. With larger datasets the performance increases, but decreases, as expected, with noisy data.

The Spearman correlation is a nonparametric measure for correlation. It does not make any assumption for the probability distribution of the data. In this study it outperforms the Pearson correlation, which can only detect linear relationships. It seems that the rank correlation is more appropriate for analyzing time series data because of its robustness against noisy data.

A crucial point in the determination of the distance measures is the chosen thresholds for discretization of the resulted matrices of continuous real values. An optimal threshold as shown in Table 2 was determined for the methods NN, GGM, all PCs, and all SCs with regard to an optimized distance measure. The other methods do not need a discretization, nice the methods provided a ternary matrix (DBN and SSM) or have already removed all nonsignificant links form the matrix (MI). A measure, independent of an artificial postprocessing of the results, is the area under the curve (AUC) score which we calculated as well. The advantage of using AUC is to obviate the need for choosing a discretization threshold. In Table 3 it is shown that a similar classification of method performances is obtained with regard to AUC and the distance measure.

Some aspects have not been addressed in this study and can be investigated further. It would be interesting to see the performances for larger networks sizes (more than 50 nodes). Some methods, such as GGM, should then show increased performances. Further, many applications are not suitable for analyzing such large datasets. For these methods a reduction of the dimension of the data has to be performed in order to obtain datasets of appropriate sizes. Different reduction methods can be investigated for that. Moreover, it would be interesting to see the impact of missing data on the reconstruction results, since in real experiments there are often not all genes included in the dataset.

It is shown that the reliable reconstruction of the whole GRN is anymore an ambitious intention and needs further progress. For that, the quality and quantity of gene expression measurements have to be improved as well as the performance of current or new algorithms. Benchmarks with realistic artificial data has to identify those methods which show the best results under different conditions.
